# Two Cases of Severe Food-Borne Botulism: Could High-Dose Methylcobalamin Accelerate Recovery?

**DOI:** 10.7759/cureus.94339

**Published:** 2025-10-11

**Authors:** Islam A Aied

**Affiliations:** 1 Accident and Emergency, Northern Lincolnshire and Goole NHS Trust-NHS, Scunthorpe, GBR; 2 Intensive Care Unit, Dr Soliman Fakeeh Hospital, Jeddah, SAU; 3 Forensic Medicine, Ain Shams University, Cairo, EGY

**Keywords:** adult botulism, forensic toxicology, mitochondria targeting therapy, neuro-critical care, neuromuscular disorders, vitamin b12 supplementation

## Abstract

Botulinum neurotoxins (BoNTs) are zinc-dependent endopeptidases that cleave presynaptic SNARE proteins and arrest acetylcholine release, producing flaccid paralysis with protracted recovery. We report two cases of a mother and son who developed severe food-borne botulism after eating Fesikh (traditional salted fish) with rapidly progressive descending paralysis requiring ventilation, prominent dysautonomia (hypotension responsive to fluids, bradycardia, dry mucosae, constipation with abdominal distension, as well as urinary retention), and intact consciousness. Both received trivalent antitoxin on day 2; the son was extubated on day 10, and the mother on day 16.

Persistent bladder atony, fatigue, weakness, and poor concentration improved with structured physiotherapy accompanied by intramuscular methylcobalamin, which was initiated on day 1 of admission and subsequently transitioned to an eight-week course of oral methylcobalamin (500 µg daily) during the post-extubation rehabilitation phase. We discuss mechanistic plausibility and hypothesize that higher-dose methylcobalamin might accelerate neuromuscular recovery by stabilizing mitochondria, attenuating caspase-mediated apoptosis, and promoting axonal regeneration and sprouting.

## Introduction

There is no doubt that botulinum neurotoxins (BoNTs) are among the most potent biological toxins. After binding to peripheral cholinergic terminals, they are endocytosed and translocate a Zn²⁺-metalloprotease light chain into the cytosol that cleaves SNARE proteins SNAP-25 (BoNT types A, E), synaptobrevin/VAMP (BoNT types B, D, F, G), and syntaxin (BoNT/C), disabling synaptic vesicle fusion and halting acetylcholine release [1]. Recovery depends on axonal sprouting and de novo synaptogenesis at the neuromuscular junction (NMJ), a slow process that underlies prolonged weakness even after antitoxin neutralizes circulating toxin [2]. In parallel, BoNTs interact with dual receptors on nerve terminals: polysialogangliosides (e.g., GT1b, GD1a) confer initial binding avidity, while protein receptors such as SV2 (for BoNT/A, E) or synaptotagmin (for BoNT/B, G) mediate internalization [3,4]. These molecular events explain both the exquisite cholinergic specificity and the prominent autonomic features in severe disease [5]. Clinically, botulism typically presents with acute cranial nerve dysfunction (e.g., diplopia, dysarthria, dysphagia), followed by a symmetric, descending flaccid paralysis that can progress to respiratory failure requiring ventilatory support. Even with antitoxin administration, there are no approved therapies to actively promote the slow process of axonal sprouting and NMJ regeneration. This therapeutic gap highlights the need for adjunctive neuroprotective or neuro-regenerative strategies, within which methylcobalamin may have potential.

Additionally, and beyond its medical relevance as a naturally occurring foodborne, wound, or iatrogenic disease, botulism has been recognized as a high-priority bioterrorism threat because of the extraordinary potency of BoNT. It is considered among the most lethal substances known, with estimated human lethal doses in the nanogram range, and the relative ease of production and aerosolization [6]. This dual identity, both as a natural disease and a potential weapon of mass disruption, underscores the urgent need for a deeper understanding of its molecular mechanisms and possible neuroprotective interventions. 

## Case presentation

Case 1

A 54-year-old mother presented to our accident and emergency (A&E) department around 12 hours following a traditional homemade fish meal (Fesikh), which was stored for more than one year in a strict anaerobic condition. She started to experience nausea, vomiting once, abdominal distension, blurred vision, ptosis, and diplopia, followed by dysphonia and dysphagia, then collapsed on a stairway. She sought medical advice at a medical facility and a polyclinic, which suspected that she had a cerebrovascular stroke or viral encephalitis, but brain imaging came back normal, as well as the blood investigations, and she was discharged despite progressive hypotonic paralysis.

On arrival to our emergency department, she was fully conscious, with a Glasgow Coma Scale (GCS) of 15/15, but bilateral descending hypotonia was rapidly complicated by failing respiratory effort with rising PCO₂ and falling oxygen saturation, mandating endotracheal intubation for respiratory support. Initial hemodynamics showed low blood pressure that responded to intravenous fluids; no vasopressors were required. Non-neurological/autonomic features were prominent in bradycardia, dry mucous membranes, constipation, abdominal distension with sluggish bowel sounds, and urinary retention. The lady’s obesity prolonged the mechanical ventilation. 

Case 2

An 18-year-old male patient who shared the same fish meal with his mother was experiencing very similar neuromuscular and autonomic symptoms and presented with her to our ER at the same time. The young man was deteriorating more quickly, particularly because severe and rapidly progressive bilateral hypotonia with rapid involvement of respiratory muscles and subsequent acute respiratory failure ended up with endotracheal intubation and mechanical ventilation. Both patients were transferred to the intensive care unit.

All routine laboratory investigations, including complete blood count, renal and liver function tests, serum electrolytes, and inflammatory markers, were within normal limits throughout the course of illness in both cases. Advanced diagnostic tests, such as toxin bioassay or electromyography, were not available locally and were not pursued, as the clinical presentation was pathognomonic of foodborne botulism and required immediate therapeutic intervention.

Treatment

Given the characteristic history, clinical examination, and the group presentation, a clinical diagnosis of food-borne botulism was made in the absence of on-site laboratory confirmation. On day 2, both patients received three doses of trivalent equine botulinum antitoxin. Nutrition was delivered via a nasogastric tube, but methylcobalamin was given via intramuscular injection starting day 1. Hypotonia improved gradually. The son was extubated on day 10; the mother on day 16. Post-extubation, both experienced prolonged bladder atony, marked fatigue, weakness, exhaustion, and poor concentration, consistent with post-botulism syndrome. A structured physiotherapy programme was initiated together with oral methylcobalamin 500 µg daily for eight weeks. They were discharged three weeks after presentation with minimal residual fatigue and ongoing outpatient rehabilitation. 

It is worth mentioning that both patients were previously healthy adults with no relevant medical or social risk factors.

## Discussion

These two linked cases underscore the diagnostic challenge when initial neuroimaging is normal and laboratory confirmation is unavailable, and they highlight how the history of an anaerobically stored salted fish was decisive. The clinical course, rapid cranial neuropathies, descending paralysis with ventilatory failure but preserved cognition, prominent dysautonomia, and sluggish post-extubation recovery, is typical for BoNT poisoning. Despite timely antitoxin, recovery depended on biological repair at the NMJ.

Additionally, prolonged mechanical ventilation was tried to be avoided from the start through quick antidote administration, daily sedation vacation, physiotherapy, high-dose methylcobalamin, and early weaning plans. There was no need to use neuromuscular blockers at any stage because of the severity of hypotonia induced by the BoNT itself; however, it was too difficult to wean them before the aforementioned time plan.

Possible mechanism of cobalamin therapy in botulism

**Figure 1:**
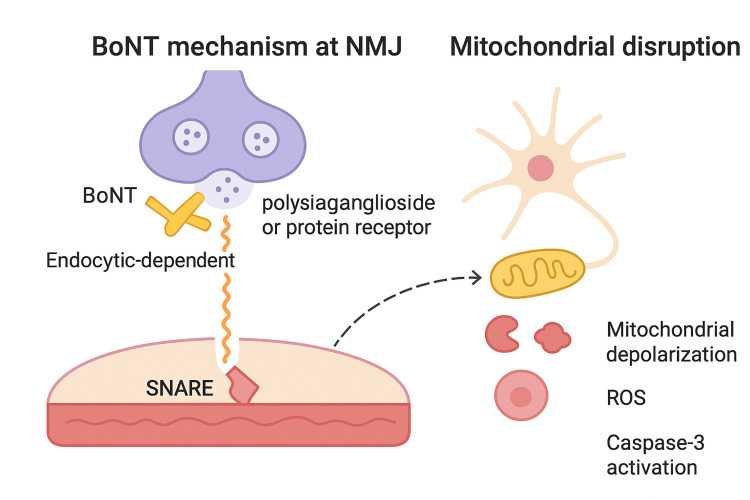
Mechanism of botulinum neurotoxin (BoNT) at the neuromuscular junction (NMJ) and downstream mitochondrial disruption. BoNT binds to polysialoganglioside or protein receptors, enters by endocytosis, and cleaves SNARE proteins, leading to synaptic transmission block. Downstream effects include mitochondrial depolarisation, reactive oxygen species (ROS) generation, and caspase-3 activation. This figure has been created by the author.

The zinc-dependent endopeptidase activity of BoNT not only arrests synaptic transmission by cleaving SNARE proteins but also induces downstream trophic disruption and neuronal degeneration [1]. Synaptic silencing diminishes activity-dependent trophic signalling and is associated in experimental systems with mitochondrial depolarisation, ROS generation, cytochrome-c release, and caspase-3 activation, suggesting engagement of the intrinsic apoptotic pathway [2]. Clinically, widespread autonomic dysfunction, including hypotension and bradycardia, is explained by BoNT action at autonomic cholinergic synapses (sympathetic ganglia and parasympathetic efferents), where SNARE cleavage reduces neurotransmitter release; reduced sympathetic vasoconstrictor output plausibly contributes to low blood pressure in severe cases (Figure 1) [3]. Methylcobalamin is the active cobalamin cofactor for methionine synthase, sustaining S-adenosyl-methionine-dependent methylation of nucleic acids and proteins. In neuronal systems, supraphysiological methylcobalamin promotes neurite outgrowth and axonal sprouting, enhances myelin basic protein synthesis, and up-regulates regeneration-associated pathways [4].

Several mechanisms are relevant to BoNT injury. First, methylcobalamin stabilises mitochondrial function, improving electron-transport efficiency and ATP generation while reducing ROS, thereby limiting mitochondrial-triggered caspase activation [5]. Second, by supporting methylation capacity, it facilitates local protein synthesis at regenerating terminals, potentially expediting SNARE protein turnover and synaptic vesicle cycling once newly sprouted boutons form. Third, methylcobalamin may interact with ganglioside-dependent repair: GM1 and related gangliosides are implicated in axonal growth and BoNT binding dynamics; enhancing membrane lipid metabolism and axolemmal integrity could aid reinnervation [6,7].

**Figure 2:**
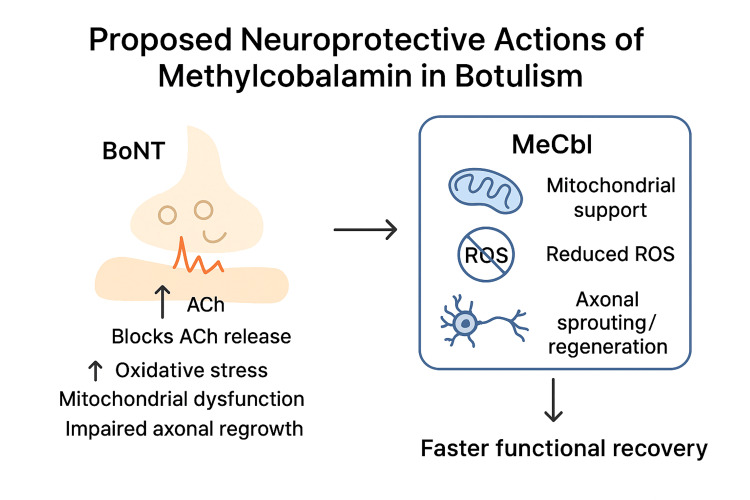
Role of methylcobolamin (MeCbl) as a neuroprotective in botulism This figure has been created by the author. ROS: reactive oxygen species

Taken together, these actions provide a biologically plausible framework in which higher-dose methylcobalamin might accelerate NMJ reinnervation and shorten clinical recovery, even though definitive clinical evidence in botulism is not yet available (Figure 2). The addition of 500 µg of methylcobalamin daily for eight weeks coincided with steady functional gains; while the dose used here was in the microgram range, the mechanistic rationale and experience from neuropathies and ALS trials suggest that milligram-level (“high-dose”) regimens merit formal evaluation for potential time-to-reinnervation benefits [8]. Finally, the bioterrorism relevance of BoNT, as listed by the CDC, highlights the need for awareness and preparedness for both natural and deliberate exposures [9].

## Conclusions

Food-borne botulism can produce severe, prolonged neuromuscular and autonomic dysfunction even with optimal care. Mechanistic data on SNARE cleavage, zinc-dependent proteolysis, mitochondrial stress, and caspase activation offer testable targets for adjunctive neuroprotective therapy. Methylcobalamin is a biologically plausible candidate to accelerate NMJ reinnervation by stabilising mitochondria, attenuating apoptosis, and promoting axonal sprouting.

Our two cases, with careful documentation of autonomic features, extubation timing, and functional gains during an eight-week methylcobalamin course, provide a clinically grounded rationale to pursue prospective evaluation. This case highlights a possible supportive role of methylcobalamin in recovery from botulism, but the association remains observational. Therefore, these findings should be considered hypothesis-generating, warranting further exploration in well-designed controlled studies.
